# Mice survival in a two-hit model of sepsis depends on intratracheal *P. aeruginosa *bacterial load

**DOI:** 10.1186/cc14062

**Published:** 2014-12-03

**Authors:** D Restagno, J-M Bonnet, A Kodjo, F Venet, C Paquet, L Freyburger, V Louzier

**Affiliations:** 1 VetAgro sup, campus vétérinaire, Marcy l'Etoile, France; 2 Groupement Hospitalier Edouard Heriot, Laboratoire d'immunologie cellulaire, Lyon, France

## Introduction

Sepsis is a systemic reaction in the presence of an infection characterized by an early systemic inflammatory response syndrome followed by a compensatory anti-inflammatory response syndrome. The first side of this typical immune response is responsible for widespread damage organ whereas the second part leads to an immunoparalysis increasing patients' susceptibility to secondary infections. The goal of this study is to finalize a murine model of sepsis to understand the physiopathology of sepsis and eventually test new therapeutic approaches.

## Methods

Male C57Bl6 mice (20 to 25 g, 6 to 8 weeks) were used. Sepsis is induced by a mild CLP (survival ≃ 80%). Cecum is isolated, ligated (1/3), and punctured twice (21G needle). A laparotomy is performed without ligation or puncture. Blood is collected 2 hours, 6 hours, 1, 2, 3, 5 and 13 days after the CLP. Plasmatic cytokines (TNFα, IL-6, IL-10) are evaluated via the Luminex technique. Five days after CLP, splenocytes are collected and used for immunological assays. Mice are intratracheally instilled with *P. aeruginosa *5 days after CLP (5 × 10^6^, 2 × 10^7 ^and 10^8 ^CFU) to evaluate survival.

## Results

Circulating TNFα, IL-6 and IL-10 levels increase 2 hours after CLP and remain high until the end of the experiment, as compared to Sham operated mice. Five days after CLP, total T cells (T-CD3^+^) population and proliferation significantly decrease as compared to the nonseptic condition whereas the T-reg cell (T-CD4^+^/CD25^+^) population significantly increases whereas it remains low in the Sham operated animals. TNFα induction by LPS in cultured splenocytes increases both for CLP and Sham operated mice but remains lower in septic mice. Survival following a secondary infection decreases while the quantity of instilled *P. aeruginosa *increases. No mortality is observed for 5 × 10^6 ^CFU, 50% of CLP die with 2 × 10^7 ^CFU and 100% of mortality is observed for both CLP and Sham with 10^8 ^CFU. Two days after instillation, *P. aeruginosa *is detected in the lungs of CLP and Sham but in the spleen and liver only for CLP mice.

## Conclusion

This study demonstrates that immunosuppression following CLP increases mice susceptibility to secondary *P. aeruginosa *intratracheal infection. However, this susceptibility depends on the bacterial load.

